# Homocysteine and copper ions: is their interaction responsible for cardiovascular-related damage?

**DOI:** 10.1007/s00726-021-03010-x

**Published:** 2021-05-29

**Authors:** Dimitrios Tsikas

**Affiliations:** Institute of Toxicology, Core Unit Proteomics, Hannover Medical School, Carl-Neuberg-Str. 1, 30625 Hannover, Germany

Dear Editor,

I read with great interest the paper by Gupta et al. “Identifying a role for the interaction of homocysteine and copper in promoting cardiovascular-related damage”, which was recently published in *Amino Acids* (Gupta et al. 2021). Homocysteine, i.e., specifically plasma total homocysteine, is considered to be associated with oxidative stress and to be an independent risk factor in over 100 conditions, especially including diseases associated with the cardiovascular and central nervous systems, and to be a guide for the prevention of disease (Smith and Refsum 2021). Patients with cardiovascular diseases (CVD) were found to have elevated total homocysteine plasma concentrations and those with levels above 20 µM had a 4.5-fold greater risk of dying than those with total homocysteine plasma concentrations below 9 µM (reviewed by Smith and Refsum 2021). Yet, the mechanisms of its actions are still incompletely explored. Many clinical studies aiming to decrease oxidative stress revealed effective reduction of the concentration of biomarkers of oxidative stress including plasma total homocysteine by B vitamin supplementation notably folate, yet without appreciable clinical improvement, or even being detrimental (reviewed by Giustarini et al. 2009; Smith and Refsum 2021).

In healthy humans, plasma total homocysteine (10 µM) consists of free l-homocysteine (0.2 µM), symmetric l-homocysteine disulfide (l-homocystine, 1.9 µM), asymmetric disulfide of l-homocysteine with albumin and other circulating proteins (Mansoor et al. 1992), and free l-homocysteine thiolactone (about 60 nM) (Arora et al., 2014). l-Homocysteine thiolactone is more reactive than l-homocysteine. For instance, l-homocysteine thiolactone can react with the terminal *N*^ε^-amine group of l-lysine residues in proteins. This post-translational modification is considered to induce vascular damage and to play major roles in many biological systems (Esse et al. 2019).

It is still unclear which homocysteine species is the culprit mainly responsible for the detrimental effects of homocysteine. In clinical studies, plasma total homocysteine is the primarily measured biomarker. In in vitro studies, free l-homocysteine is used to investigate possible biological effects and underlying mechanisms assumed to be exerted by homocysteine. Yet, effects are seen at l-homocysteine concentrations far exceeding those of free L-homocysteine and even those of plasma total homocysteine. It seems that free l-homocysteine is a species with very weak biological activity.

Based on previous observations by many groups, Gupta and colleagues investigated in vitro the combined effects of free l-homocysteine and Cu^2+^ added as CuCl_2_. Interestingly, l-homocysteine, but not its homologue l-cysteine (Cys) or the Cys-containing tripeptide glutathione (GSH) were found to interact with Cu^2+^. The authors observed an intramolecular hydrogen atom (H) transfer resulting in α-amino carbon-centered radical, which is known to promote biological damage (see Refs. in Gupta et al. 2021). Critical concerns in the experiments by Gupta et al. (2021) are the drastic experimental conditions, i.e., reflux for 2 min and the use of very high concentrations of l-homocysteine (5 mM) and of CuCl_2_ (5 mM). Such concentrations are almost three orders of magnitude higher than pathophysiological and even toxic concentrations of l-homocysteine (Mansoor et al. 1992) and CuCl_2_. Indeed, CuCl_2_ is toxic and lethal (e.g., LD_50_ 584 mg/kg in the rat). Such a dose would lead to blood Cu^2+^ concentrations of about 50 mM in laboratory (200 g weighing) rats. Also, no demonstration of an l-homocysteine/CuCl_2_-specific biological damage was reported, except for the observation of a *C*^α^ reactive radical (Gupta et al. 2021). It is worth mentioning that Cu^2+^ ions can bind to circulating proteins, such as albumin and enzymes, such as ceruloplasmin, the most abundant carrier of Cu^2+^ ions in the human circulation. At physiological concentrations, ceruloplasmin can oxidize nitric oxide (NO) to nitrite, thus attenuating its vasodilatatory and antiaggregatory effects in the circulation. This could be a Cu^2+^ ions involving mechanism possibly responsible for cardiovascular disease (Shiva et al. 2006).

Free L-homocysteine and free Cu^2+^ ions are unlikely to play a major role in cardiovascular disease, even not in severe hyper-homocysteinemia (> 15 µM), by inducing α-amino carbon-centered radical formation from l-homocysteine (Gupta et al. 2021). Reported observations obtained using high concentrations of l-homocysteine and of potentially important factors such as Cu^2+^ ions are misleading and presumably not related to homocysteine’s biology. A possible corrective could be to measure all circulating homocysteine forms in plasma. Another mandatory corrective could be the use in in vitro experiments homocysteine-species concentrations found in diseases, the use of homocysteine-species doses leading to pathologically, but not to toxicologically relevant plasma concentrations of homocysteine species.
